# ‘Fostering the Future’: Exploring Barriers and Enablers to Doctors Pursuing a Clinician Educator Pathway in Obstetrics and Gynaecology

**DOI:** 10.1111/ajo.70047

**Published:** 2025-05-28

**Authors:** Sarah Van Der Hock, Naomi Holbeach, Edwina Coghlan

**Affiliations:** ^1^ Melbourne Medical School University of Melbourne Melbourne Victoria Australia; ^2^ Department of Obstetrics and Gynaecology Joan Kirner Women's and Children's Hospital – Western Health Melbourne Victoria Australia; ^3^ Department of Obstetrics, Gynaecology, and Newborn Health University of Melbourne Melbourne Victoria Australia; ^4^ Mercy Hospital for Women Melbourne Victoria Australia

**Keywords:** barrier, clinician educator, enabler

## Abstract

The Royal Australian and New Zealand College of Obstetricians and Gynaecologists (RANZCOG) is committed to developing a sustainable, high‐quality clinical education workforce. To train exceptional doctors, exceptional educators are needed. In order to better understand the barriers and enablers to careers as a clinician‐educator in Obstetrics and Gynaecology, further research related to the educator experience is required. We argue that renewed efforts to address these barriers and enablers are needed to support Obstetricians and Gynaecologists with expertise and passion for teaching, as they contribute significantly to the training and sustainability of the current and future medical workforce. Those supports should target the following areas identified from this literature review: institutional support and culture, personal motivation and interest, balance of clinical service and delivery of education and career impact and advancement. Amid rising numbers of medical students, post‐pandemic challenges in the hospital and workforce pressures for specialists and general practitioners in Australia, it is essential to prioritise clinical education strategic planning and research. This review contributes to the literature by supporting existing efforts to develop structured educator pathways such as the new RANZCOG Academy of Clinician Educators.

## 1 Introduction

Clinician educators (CEs) play a vital role in educating the future generations of specialists and women's health practitioners throughout Australia. Historically, the notion of altruism and ‘paying it forward’ has driven teaching of the next generation of clinicians [1]. Internationally, the term clinician educator (CE) lacks consensus. The UK‐based Academy of Medical Educators defines a CE, as a practitioner ‘who has committed a significant amount of their time, energy and professional development to medical education and can demonstrate that this has become an important component of their career’ [2]. The field has been in part, professionally recognised through the development of clinical schools, dedicated societies and journals and medical education conferences. In Australia, organisations such as the Australian Medical Council (AMC), the Australian and New Zealand Association for Health Professional Educators (ANZAHPE) and the Medical Deans Australian and New Zealand (MDANZ) play key roles in the development and promotion of clinical educators. Despite the work of these organisations, there remains a gap in formal recognition and support for clinician educators. In a large global survey of almost 300 educators, 40% of clinical educators felt recognition of their involvement in medical education is low when compared to their clinical work and research [2, 3]. Medical education has often been seen as complementary to one's primary specialty and undertaken via altruism, loyalty and duty, and at times in an *ad hoc* fashion with varying levels of quality [4].

Until recently, CEs did not hold formal affiliation or governance within their own specific specialty colleges in Australia. Consequently, there was no standardised system for providing assessment and feedback or professional development on their teaching performance [5]. Royal Australian and New Zealand College of Obstetricians and Gynaecologists (RANZCOG), Australasian College for Emergency Medicine (ACEM) and Royal Australian College of General Practitioners (RACGP) are some of the recent colleges to endorse working groups, governance and advocacy for this important group of fellows. RANZCOG has recently launched the new Academy of Clinician Educators (ACE), an initiative designed to support, develop and recognise clinician educators among the specialty.

The undersupply of Obstetricians and Gynaecologists, combined with projected workforce shortages in the near future, poses further burdens on the training needs of our increasing number of medical students and junior doctors [6]. Given the growing need for skilled educators within Obstetrics and Gynaecology, it is crucial to understand the factors that influence a career in clinical education. A narrative review approach was chosen to comprehensively synthesise existing qualitative research, identify key themes and inform future strategies. To the best of our knowledge, this is the first narrative review to focus on the clinical educator experience in Obstetrics and Gynaecology.

## 2 Aim

To review and synthesise the current literature on the barriers and enablers influencing clinicians' pursuit of a clinician educator pathway amongst Obstetricians and Gynaecologists in Australia.

## 3 Methodology

Initially, two databases (1) Ovid and (2) Web of Science were searched from January 2013 to August 2024 using search terms comprising of ‘barrier’, ‘enabler’, ‘motivation’, ‘clinician educator’, ‘medical educator’, ‘medical teacher’, ‘women's health’, ‘obstetrics’ and ‘gynaecology’. Wildcards were applied to account for varied forms of the keywords. As discussed in the introduction, we recognise that definitions in the field vary, and while ‘clinician educator’ was the primary focus of our narrative review, other related terms were considered to broaden the search scope.

Initially, the research question was specific to Obstetrics and Gynaecology in Australia; however, only one paper met the inclusion/exclusion criteria. The field was thus broadened to encompass any medical speciality internationally.

Inclusion criteria:

1. Full‐text English;
2. Published in the previous decade to ensure relevancy;
3. Clinician educator of any medical specialty (not limited to Obstetrics and Gynaecology).

Exclusion criteria:

1. Education that was not of a doctor or medical student (i.e., nursing or allied health education);
2. Clinician educators were not a doctor with a medical degree;
3. The focus was not on the enablers and barriers (i.e., structure of medical school, education programme, evaluation of an established education programme);
4. The paper did not have a study aim or formal methodology.

The initial search yielded 337 papers (Ovid 191, Web of Science 146). 102 duplicates were removed, leaving 235 papers for title and abstract screening for eligibility by two researchers EC (clinician educator) and SV (learner). Reflexivity was considered throughout the literature search, as both authors bring different perspectives to the study being a clinician educator and a learner.

75 papers remained for full‐text screening, including assessing for quality of evidence. Seven papers were included for review following full‐text stage. Citation searching of the final articles was conducted, yielding a further four papers. In total, 11 papers were included for analysis from the two databases. During the review stage, two additional databases (3) BMC Medical Education and (4) BMJ Medical Education were searched using the same date range and search terms. These databases were selected for its exclusive focus on medical education, allowing for a more targeted review. An additional 10 papers underwent full‐text screening, of which one met the inclusion and exclusion criteria, leaving 12 papers for review. Qualitative analysis was conducted manually, drawing upon Braun and Clarke's six stage thematic analysis technique [7].

## 4 Results

Initial thematic analysis of the 12 papers (see Table 1) identified 26 barriers and enablers which were further categorised into four overarching themes: (1) institutional support and culture, (2) personal motivation and interest, (3) balance of clinical service and delivery of education and (4) career impact and advancement. These are summarised in Table 2.

**TABLE 1:** Summary of the included papers.

Study title	Type of study	Study characteristics—country, date, medical specialty	Barriers	Enablers
Assessment of burnout and associated factors amongst medical educators [8].	Mixed methods	Pakistan, 2018–2019, clinician‐educators but no further specialty specified	Key barriers included: (1) high levels of burnout, (2) lack of identity, (3) lack of recognition of department by faculty, (4) poor job structure, (5) no formal mentoring, (6) tired of justifying job title to other departments Most respondents had moderate 71 (70.3%) level emotional exhaustion whereas 9 (8.9%) had severe level. Average level of depersonalisation was seen in 73 (72.3%) and severe level was observed in 20 (19.8%) respondents	N/A
Becoming a medical educator: motivation, socialisation and navigation [4]	Qualitative	Australia, 2014, junior doctors completing medical education registrar (MER) post	Key barriers included: (1) lack of clarity of job title and poor career direction, (2) lack of understanding of their role by peers and senior clinicians, (3) feeling guilty about having dedicated workspace and lunch compared to full‐time clinicians, (4) strong pull back to full‐time clinical work or research, (5) the need for research and lacking confidence to undertake it	Key enablers included: (1) negative learner experience, (2) excellent and inspiring teachers and/or mentors, (3) supervisors who had adequate time and knowledge, (4) improved work–life balance
Developing a workplace‐based learning culture in the NHS: Aspirations and challenges [9]	Qualitative	England, 2020, physicians	Key barriers included: (1) feeling undervalued in their role, (2) under‐resourced workforce with poor teaching facilities, (3) inaccurate job planning, (4) issues with middle management, (5) absence of formal measures of progress and feedback, (6) clinical targets prioritised over delivery of education, (7) time poor	Key enablers included: (1) integrating teaching into working practices, (2) shared vision for education and institutional culture of learning, (3) presence of mentors and supervisors
‘It was serendipity’: a qualitative study of academic careers in medical education [10]	Qualitative	Australia, 2015, clinician‐educators but no further specialty specified	Key barriers included: (1) the need for research, (2) lack of financial compensation, (3) invisibility of medical education, (4) lack of visible training pathway or promotion opportunities	Key enablers included: (1) diversity of professional identity, (2) relationships with students, (3) fulfilling higher purpose of training future doctors, (4) perception of serendipity, (5) early experiences of being a teacher, (6) excellent mentors, (7) negative learner experience
Professional Identity Formation of Medical Educators: A Thematic Analysis of Enabling Factors and Competencies Needed [11]	Qualitative	India, 2022, clinician‐educators but no further specialty specified	N/A	Key enablers included: (1) availability of supportive and opportunity‐driven workplace, (2) continuous training through faculty development programs, (3) strong networking and professional circles, (4) motivational mentors and role models, (5) negative learner experience
How can clinician‐educator training programs be optimised to match clinician motivations and concerns? [5]	Qualitative	Canada, 2015, physicians	Key barriers included: (1) decreased productivity in the clinical environment and therefore reduced salaries, (2) increased length of day, (3) lack of financial compensation, (4) patient concerns and ethical issues of student presence, (5) lack of clinician‐educator confidence	Key enablers included: (1) enjoyment in observing outcomes of teaching, (2) appreciation for new perspectives gained from students, (3) sense of duty, (4) self‐reflection on own skills, (5) opportunity for teaching awards and University appointments
Exploring the tensions of being and becoming a medical educator [12]	Qualitative	Scotland, 2017, clinician‐educators but no further specialty specified	Key barriers included: (1) loss of identity if one chooses to be a full‐time educator, (2) feeling undervalued, (3) training pathways and tracks to promotion less defined, (4) lack of financial compensation, (5) lack of mentors, (6) the need for research and unavailability of PhD programs, (7) no formal mentoring, (8) low clinician‐educator confidence, (9) difficulty balancing teaching with clinical workload	N/A
Becoming outstanding educators: What do they say contributed to success? [1]	Qualitative	Canada, 2015, internal medicine, surgery, or paediatrics	N/A	Key enablers included: (1) perception of serendipity leading to first opportunities and taking initiative in saying ‘yes’, (2) professional networks and social contacts, (3) continuous training through faculty development and programs, (4) presence of mentors, (5) supportive work culture, (6) intersection of clinical and educator identities/diversity of professional identity
Influences on and characteristics of the professional identity formation of clinician educators: a qualitative analysis [13]	Qualitative	USA, 2016, wide range of specialities including medical and surgical	Key barriers included: (1) the need for research, (2) lack of formalised training program, (3) perceived lack of credibility	Key enablers included: (1) strong mentorship program, (2) presence of inspiring role models, (3) early experiences of wanting to be a teacher, (4) naturally gifted in teaching, (5) being responsive to the learner, (6) perception of serendipity, (7) sense of duty
Key factors in work engagement and job motivation of teaching faculty at a university medical centre [14]	Qualitative	USA, 2021, wide range of specialities including medical and surgical	Key barriers included: (1) unmotivated students, (2) poor teaching facilities, (3) time poor, (4) bureaucratic issues, (5) monotonous group teaching, (6) low clinician‐educator confidence	Key enablers included: (1) ability to teach their own specialty, (2) noticeable appreciation for teaching from students, (3) feedback, (4) motivated when there was clear organisation, expectations and time planning (5) institutional recognition and support
‘Why physicians teach: giving back by paying it forward.’ [15]	Mixed methods	Netherlands, 2013, 12 hospital‐based specialities	Key barriers included: (1) lack of financial compensation	Key enablers included: (1) teaching as an integral part of identity, (2) teaching allowed them to repay former teachers for their own training, (3) fulfil higher purpose and contribute to development of future doctors, (4) teaching as a form of learning, (5) personally energising and gratifying, (6) negative learner experience
Understanding barriers, enablers and motivational factors for Australian healthcare educators teaching university students on clinical placement using the validated Physician Teaching Motivation Questionnaire [16]	Mixed methods	Australia, 2024, wide range of specialities, all participants were affiliated with Deakin University's School of Medicine	Key barriers included: (1) competing work requirements, (2) time management, (3) income expectations, (4) IT requirements, (5) requirements of the HR onboarding process and (6) requirements of ongoing compliance	Key enablers included: (1) continuing professional development, (2) access to teaching material, (3) assistance with research, (4) library access and (5) a sense of altruism

**TABLE 2:** Core themes mapped onto overarching concepts as to why doctors pursue medical educator pathways.

	Enabler	Barrier
Institutional support and culture	Organisational culture of teaching and opportunity‐driven workplace	Undervalued (financially) and poor academic recognition among colleagues Competing responsibilities and lack of protected time Unclear job description and lack of visible training program Lack of financial compensation Poor workforce resourcing and teaching facilities Lack of mentors and supervisors The need for robust measures of success and formalised feedback Bureaucratic issues Onboarding HR requirements and ongoing compliance
Personal motivation	Developing relationships with students Learner experience Sense of duty Naturally ‘gifted’ and early passion for teaching Teaching as a form of personal learning Work‐life balance	Lack of financial compensation Undervalued (financially) and poor academic recognition amongst colleagues Lack of clinician‐educator confidence High levels of burnout
Balance of clinical service and delivery of education	Diversity of professional identity Sense of duty	Competing responsibilities and lack of protected time High levels of burnout Perceived decreased productivity in the clinical setting Patient concerns/ethical issues with student in the clinical setting
Career impact and advancement	Influential role models Diversity of professional identity Learner experience Serendipitous Networking opportunities Assistance with research Library access	Unclear job description and lack of visible training program Lack of education‐focused metrics for academic promotion Lack of mentors and supervisors The need for robust measures of success and formalised feedback

### 4.1 Institutional Support and Culture

A key enabler found in almost half of the papers referenced the importance of having a good organisational culture of learning, and a supportive and opportunity‐driven workplace environment [4, 9, 11, 13, 15].

Barriers related to institutional support included the absence of a training pathway or standardised job description [4, 8, 10, 12, 14]. Medical education lacks visibility as a practice and consequently CEs became increasingly tired of justifying their job title and role to colleagues in other departments. Without a clearly defined educator career track, there is little opportunity for formalised feedback [4, 5] or mentoring programmes [8, 12]. One‐third of the studies cited CEs reported being financially disadvantaged when compared to their full‐time clinical counterparts [1, 5, 10, 12]. One study referenced the onboarding HR requirements and ongoing compliance, such as course meetings, as a barrier [16].

### 4.2 Personal Motivation

Just under half of the included studies focused on personal inspiration from their own experience of teaching during medical school, or from interactions with informal mentors who shared a similar interest in medical education [4, 9, 10, 13, 14]. Interestingly, CEs were equally motivated by their own personal negative experiences as a learner as they were by positive learner experiences [1, 4, 10, 11]. Positive relationships developed between the educator and learners were also noted as a key enabler in just under half of the papers [1, 5, 10, 14, 15].

Time pressures and juggling competing responsibilities of clinical work, research and teaching without protected time was a significant a barrier in just under half of the literature [4, 8, 9, 12, 15]. CEs faced challenges scheduling formal teaching sessions, and provided insights into doing it before and after clinical sessions, during their lunch break and as part of their protected clinical support time. These competing demands and lack of protected time were noted to contribute to feelings of burnout [8]. The absence of clinician‐educator training and professional development and perceived low confidence in one's own teaching ability was listed as a barrier in nearly one‐third of the literature [5, 12, 15].

### 4.3 Balance of Clinical Service and Delivery of Education

Historically, a reliance on CE's goodwill and sense of duty to pass knowledge onto future generations has driven the field of medical education. This reliance on the altruistic dedication of CEs is a key enabler in four out of the 11 papers [1, 5, 10, 14]. Furthermore, the multifaceted professional identity of a CE that incorporates educational responsibilities with clinical practice and research is a crucial enabler [1, 4, 10, 13].

One article discussed the clinicians' perceived decreased productivity in the clinical setting when teaching simultaneously and the further possible financial disadvantage as patient quotas may not be met [5].

### 4.4 Career Impact and Advancement

The strongest enabler emphasised the positive influence of role models and mentors who served as inspiration throughout medical school, training and post‐fellowship. Positive mentoring provided CEs crucial guidance and support in their development as a ce [ 4, 9, 10, 13, 14]. Access to institutional libraries and dedicated research support services was identified as a key enabler, facilitating clinician educators' engagement with evidence‐based teaching and scholarly activities [16].

CEs expressed frustration with the lack of reproducible metrics for recognition and promotion compared with academia and research, especially for those whose expertise and interest lied as an educator, which is described in almost half of the 11 papers [4, 10, 12, 14].

## 5 Discussion

In 2022, the Medical Schools Outcome Database published that 82.6% of final year Australian medical students were interested in teaching as part of their future career [17]. Despite the strong interest expressed by Australian graduates, University teaching centres continue to face challenges in filling senior teaching positions with dedicated and skilled CEs [2] and few hospitals have dedicated fractional appointments for the purpose of education.

Our review found the lack of institutional support and recognition of educators in workplace culture are the most common barriers to pursuing a career as a CE. Previous literature has documented the correlation between the quality of teaching and quality of care patients receive [18]. When clinical service and education are viewed as mutually exclusive, staff morale, retention of high‐quality CE and consequently training and patient care may be subpar [19].

A core challenge faced by CEs is the competing demands of clinical service and research with the delivery of education to the next generation of doctors. A 2020 survey‐based study of fellows in a variety of subspecialty training programmes in the United States found that 87% believed being an educator was moderately or extremely important to their future career plans [20]. Despite this overwhelming interest, CEs were only able to devote a negligible amount of time towards education, referencing a lack of protected time and a strong pull‐back to clinical service as crucial hurdles [21]. The current literature shows the need for research output to advance one's education career as a major obstacle, combined with a lack of reproducible metrics for CEs. A current barrier to career advancement for CEs is the potential requirement for medical education research and higher postgraduate studies for the attainment of university promotions. As the focus of the tertiary institutions and our hospitals moves towards research, medical schools must return to their primary purpose of education. This essential realignment is also recognised by RANZCOG, which lists education as one of its core organisational values [22].

To overcome barriers recognised in the literature, we offer three recommendations to boost the retention rates of skilled CEs. These recommendations address six key barriers: inadequate academic recognition, unclear job descriptions and the absence of a visible training programme, lack of mentors and supervisors, the absence of formalised feedback, insufficient clinician‐educator confidence and the lack of education‐focused metrics for academic promotion. First, the introduction of a criteria‐led educator portfolio (EP) is recommended. While the concept of an EP is widely utilised in other industries such as engineering, EPs in medicine can vary widely in content and are difficult to assess due to the lack of standardised evaluation guidelines [23]. The implementation of structured EPs would provide an objective summary of acts of service to education, such as the number of lectures and bedside tutorials provided, colleague and student feedback, curriculum redesign involvement and education research [24]. It will acknowledge merit is awarded not only on the output of medical education research but also on the delivery of teaching on the floor, a barrier identified in this review. Second, a clear definition of a CE endorsed by the Australian Medical Council (AMC) is required in Australia. The current ambiguity in the definition of a CE may give the impression education is merely a requirement of routine professional activity, rather than a much‐needed formal career pathway [2]. Without a nation‐wide consensus of the clear roles, responsibilities and attributes required to be a CE, aspiring educators may be unsure of how to progress their career within university medical centres [25, 26]. Finally, the introduction of a standardised CE career pathway or medical education fellowships in Australia should be considered. Specialty colleges may draw inspiration from the Canadian CanMeds Clinician Educator Program [27], which is the first structured national CE programme, or medical education fellowships in the United States in specialities such as Emergency Medicine. A review of post‐residency medical education fellowships in the United States demonstrated both institutional and individual benefits; however, almost half of the fellowships did not include formal evaluation or lacked reproducible metrics [28], a barrier also identified in our review.

With structured fellowships and standardised pathways, additional challenges related to lack of a visible training pathway, absence of formal mentoring programmes and lack of measures of success and methods of formalised feedback may be addressed. RANZCOG has already taken steps towards formalising clinician educator training through the implementation of the Medical Education Advanced Training Module (ATM) [29]. This structured programme provides a comprehensive foundation in educational theory and practice, combining two Clinical Education Training components, three mandatory units and an elective completed over 12 months. Trainees are supported by an appointed Training Supervisor, receive direct supervision from a consultant obstetrician and gynaecologist and are equipped with the necessary resources to complete assessments effectively. Through this initiative, the Medical Education ATM is beginning to address several key barriers to clinician educator development identified in this review, including the need for structured pathways, supervision and institutional support.

This is the first narrative review comprehensively evaluating the key barriers and enablers to doctors pursuing a clinician educator pathway in Obstetrics and Gynaecology. A significant limitation of the review is the paucity of literature relating to the educator experience in O&G and for the Australian clinician. Second, papers included in this review were case studies and semi‐structured interviews focusing on the experience of CEs from one hospital or institution. The smaller sample size may not be representative of the entire population. Lastly, none of the papers focused on one medical specialty, instead often grouping together a wide variety of medical and surgical specialities. Certain fields encounter unique challenges not experienced by others. Hence, we recommend further research focused on the clinician educator experience in Obstetrics and Gynaecology, given the specialty's unique nature, which involves sensitive histories, examinations and the need for specialised skills in distinct clinical environments. These findings may also be applicable to other fields, particularly procedural specialties. Within RANZCOG, we welcome new initiatives such as the Academy of Clinician Educators, which supports, develops and fosters excellence among clinician educators.

## Ethics Statement

Institutional ethics committee approval was not required as this manuscript is a brief narrative review and does not involve original research or human/animal subjects.

## Conflicts of Interest

The authors declare no conflicts of interest.
